# 

**Published:** 1879-07

**Authors:** 


Photographic Illustrations of Skin Diseases, complete in twelve parts. By George Henry Fox, A. M., M. D. With forty-eight colored plates taken from life. New York: E. B. Treat, No. 805 Broadway. Part I, Price $2,00.

Diseases of the Intestines and Peritoneum. By John Lyer Bristowe, M. D., J. R. Waddell, M. D., J. W. Begbie, M. D., S. O. Habershon M. D., T. B. Curling, F. R. S., and W. H. Ransom, M. D. New York: Wm. Wood & Co. pp. 240.

Pott's Disease, its Pathology and Mechanical Treatment, with remarks on Rotary Lateral Curvature. By Newton M. Shaffer, M. D. New York: G. P. Putnam's Sons. pp. 82.

The Pharmacopœia of the British Hospital for Diseases of the Skin. By Balmanno Squire, M. D. London. J. & A. Churchill. pp. 59.

Elements of Modern Chemistry. By Adolph Wurtz. Translated and edited from the fourth French edition, by Wm. H. Greene, M. D. Philadelphia: J. B. Lippencott & Co. Buffalo: Peter Paul & Bro. pp. 676.

———·o:———

### Pamphlets Received.

Fifty-fifth Annual Announcement of the Jefferson Medical College of Philadelphia.

Sixteenth Annual Report of the New York Society for the Relief of the Ruptured and Crippled.

Announcement of the Medical Department of the University of Pennsylvania, for the One hundred and Fourteenth Session.

University of Maryland. Seventy-second Annual Circular of the School of Medicine.

Posture as a means of relief in strangulated and incarcerated Hernia. By Frank H. Hamilton, A. M., M. D.

The Radical Cure of Hernia by the Antiseptic use of the Carbolized Catgut Ligature. By Henry O. Marcy, A. M., M. D.

On the Spasmodic stricture of the Urethra. A reply to Dr. F. N. Otis. By Henry B. Sands, M. D.

Minutes of the Meeting of Organization and Proceedings of the Sanitary Council of the Mississippi Valley.

The structure of the Vessels of the Nervous Centres in Health, and their changes in disease. Part III. By Theodore Deecke.

The hand as a Curette in Post-Partum Hemorrhage. By Henry P. C. Wilson, M. D. Baltimore, Md.

The American Medical College Association. Third Annual Meeting.

"Observations on Shock." Read before the Medical Society of the State of California. By E. H. Woolsey, M. D.

Other Symptoms of Nervous Exhaustion, (Neurasthenia). By George M. Beard, A. M., M. D.

Bibliotheca Dermatologica. Piffard.

Pendulum Leverage of the Obstetric Forceps. By Albert H. Smith, M. D. Philadelphia.

Annual Announcement of the Medical Department of the University of Buffalo, for the Session of 1879–1880, with catalogue of previous session.

Eye troubles in General Practice. By Henry D. Noyes, M. D.

Second Annual Report of the Eye and Ear Department of St. Mary's Hospital, and St. Mary's Free Eye and Ear Infirmary, Detroit, Mich.

Provisional Report of the Committee of the New York Neurological Society, relative to the subject of Insane Asylum Abuses.

### INDEX.

**A**

Address, by Prof. T. F. Rochester ........ 441
Asphalt, where obtained and how used. By * - * M. D ........................... 128
Atomizer, Antiseptic. ..................... 153
Artificial Respiration. ..................... 187
Antiseptic Dressing, of Prof. Lester, ....... 61
American Medical Association. ............. 394
A New Anæsthetic, Chloramyl, ............. 140
Alcohol in Health and Disease, ............. 22
Albuminuria, Occasional non-significance of, ................................ 24
Alumni Association of Buffalo Medical College, Meeting of ......................... 318
Andrews, Judson B., M. D., Address of Before Alumni Association Buffalo Medical College, ............................ 340
American Medical Editors, Association of .. 433
Acid Chlor-acetic, in Cancer, ............... 390
Arkansas, Fourth Annual Meeting of the State Society of ......................... 436
Axles, Patent Spring Washer ............... 194
Albany, Medical Society of the County of 219 259
American Medical Association, ............. 434
American Medical Association, Fare to, .... 393
American Journal of Insanity, Libel Suit against the Editor of, ................... 151
Animal Hypophosphites Glycerite. By E. C. Lester, M. D., ....................... 13

**B**

Buffalo Telephonic Exchange ............. 354
Buffalo College of Rational Medicine, ...... 393
Buffalo Medical and Surgical Journal, Vol. xviii ................................ 33
Buffalo Medical Association, Proceedings of, ...................45, 95, 124, 247, 411
Books and Pamphlets Received,.. ......39, 80 119, 160, 200, 240, 280, 320, 358, 359, 398, 400, 439, 440, 474.

Books Reviewed.
- Fifth Annual Report of the State Commissioner in Lunacy ................ 37
- Physics of the Infectious Diseases. E. A. Logan, ........................... 38
- Congenitial Occlusion and Dilation of Lymph Channels. Samuel C. Busey,. 38
- Atlas of the Diseases of the Skin. Balmanno Squire, ....................... 39
- The Obstetric Gazette, ....... ...... 39
- The Cincinnati Lancet and Observer, .. 39
- Transactions of the American Gynecological Society .. ...... ... ...... 77
- Sources of Muscular Power. By Austin Flint, Jr., M. D., ................. 78

Books Reviewed Continued.
- A Manual of Operative Surgery. By Lewis A. Stimson, B. A., M. D., ..... 79
- Anatomy, Descriptive and Surgical, .... 118
- A New Enterprise, ...................... 119
- Fownes' Manual of Chemistry, .......... 156
- Barnes on Diseases of Women, ... .... 156
- Nervous Diseases. By Allan McLane Hamilton, M. D., ..................... 158
- Ziemssen's Cyclopedia of the Practice of Medicine, Vol. XIII .............. 196
- Playfair's System of Midwifery, ........ 198
- Elementary Quantitive Analyses. By Alexander Classen .... ........... 236
- Ziemssen's Cyclopedia, Vol. XVII, ..... 236
- On the Therapeutic Forces. By Thomas J. Mays, M. D., ...................... 237
- Transactions of Medical Society, West Virginia, .............................. 239
- Reports of the Surgeon-General, ........ 278
- Cell Doctrine. By James Tyson, M. D. 278
- Medical and Surgical Uses of Electricity. By George M. Beard, A. M., M. D., and A. D. Rockwell, A. M., M. D., ........ 279
- Diseases of the Bladder and Urethra in Women. By Alexander J. C. Skene, M. D., ................................ 279
- Contributions to Reparative Surgery. By Gurdon Buck, M. D., ............. 279
- Principles and Practice of Surgery. By John Ashurst, M. D., ............... 319
- The American Quarterly Microscopical Journal. ........ ... ... ... ...... 
- Monograph on Diphtheria. By William C. Reiter, A. M., M. D., .............. 355
- Antagonism of Therapeutics. By J. Miller Fothergill, M. D., ............. 355
- Localization of the Brain. By J. M. Charcot, .... ................. ...... 356
- Index to Original Communications in Medical Journals of the United States and Canada. Compiled by Wm. D. Chapin, .......... ................. 356
- On Rest and Pain. By John Hilton, F. R. S., F. R. C. S. ................... 357
- Medical Chemistry. By C. Gilbert Wheeler. .............................. 357
- A Guide to the Practical Examination of the Urine. By James Tyson, M. D. 357
- An Atlas of Human Anatomy. Rickman John Godlee, M. S., F. R. C. S... 358
- Practical Surgery, including Ligations and Amputations. By J. Ewing Mears, ................................ 358
- Transactions of the Medical Society, of the State of New York, ............. 358
- Transactions of the Ohio Medical Society, ................................ 358

BOOKS REVIEWED CONTINUED.
Clinical Diagnosis. By James Finlayson, M. D ........ 395
Lectures on Bright's Disease of the Kidneys. By J. M. Charcot, ........ 396
A Manual for the Practice of Surgery. By Thomas Bryant, F. R. C. S., ........ 397
Notes on the Treatment of Skin Disease. By Robert Liveing, A. M. M D. 398
A Manual of Physical Diagnosis. By Francis Delafie d, M. D., and Charles F. Stillman, M. D ........ 398
A Practical Treatise on the diseases of the Testis and of the Spermatic Cord and Scrotum. By T. B. Curling, F.R.S. 436
An introduction to Pathology and Morbid Anatomy. By T. Henry Green, M. D ........ 437
Conspectus of Organic Materia Medica and Pharmacal Botany. By L. E. Sayre, P. H. G ........ 437
A Manual of the Diseases of Children, with a formulary. By Edward Ellis, M. D. ........ 438
Differential Diagnosis. By F. De Haviland. M. D ........ 438
Modern Medical Therapeutics. By George H. Napheys, A. M., M. D ........ 438
Spermatorrhœa. By Roberts Bartholow 471
Tabular Handbook of Auscultation and Percussion. By Herbert C. Clapp, A. M., M. D ........ 472
National Dispensatory. By A. Stille and John Marsch ........ 472
A Guide to the Quantative and Quantative Analysis of the Urine ........ 473
Babcock. Heman P.,, M. D., Death of ..235, 276
Blake, George M., M. D. Suits against Surgeons, ........ 309
Barnes, Joseph K., United States Census Letter from ........ 226
Board of Health, Illinois State Attacking Quackery, ........ 184
Bladder, Treatment of Chronic Catarrh of the, and Acute Cystitis. By Theo. Deecke, 241
Bladder, Removal of a portion of a Catheter from the ........ 25
Bright's Disease, Injection of milk in ........ 464
Buffalo Medical College, Progress and Prospects of. By Prof. James P. White, ........ 321
Buffalo Medical College, Meeting of the Alumni Association of ........ 318
Buffalo Medical College, Commencement in the ........ 316

### C

CORRESPONDENCE, ........ 17
Correspondence, ........ 149
Correspondence, ........ 190
Correspondence ........ 456
Correspondence ........ 460
Counter Prescribing ........ 37
Clergyman's Sore Throat, Chronic Follicular Pharangitis. By H. S. Davis, M. D., ........ 302
Convention of Medical Colleges, Call for ........ 319
Commencement in the University of Buffalo 316
Chemical Analysis, Its importance in cases of Obscure Diagnosis. By Prof. C. A. Doremus. ........ 281
Chicago Medical College, ........ 393
Chronic Catarrh of the Bladder. By Theodore Deecke, ........ 241
Chloramyl, A new Anæsthetic and improvad Inhaler, ........ 140
Circular from Surgeon-General to Physicians and others, ........ 153
Chloral Anæsthesia in Children, ........ 183
Cancerous Disease of the Lungs. By Joseph Fowler, M. D ........ 41
Chronic Catarrh of the Bladder, Treatment of By Theodore Deecke ........ 241
Cataract, Some Practical points in Operat- Operating for ........ 381
Cervix Uteri, Amputation of ........ 373
Chronic Follicular Pharangitis. By H. S. Davis, M. D ........ 302
Chlorate Potassium, Saturated Solution, in Treatment of Ulcers. By T. M Rochester, M. D ........ 177
Cysts, Dermoid and Ovarian. By C. H. Eames, M. D ........ 98
Cells in Cystic Tumors ........ 468
" Drysdale ........ 468
Comstock, T. G., M. D. Remarks on operation for Fistula-in-ano, ........ 294
Cramp, Telegraphists ........ 29
Consumption, Syphilitic ........ 390
Cancer, Chlor-acetic Acid in ........ 390

### D

Damiana (Turnera Aphrodisiaca) ........ 273
Diphtheria, ........ 273
" Treatment of ........ 228, 385
" and Sewer Gas ........ 113
" Causes of. By Henry Mills, ........ 366
Dialyzed Iron ........ 181, 394
Diarrhea of Children and its Treatment, ........ 65
Death under Ether, ........ 24
Decimal System of Notation, ........ 30
Deecke, Theodore, on the Treatment of Catarrh of the Bladder, ........ 241
Death of Dr. Waddel, ........ 394
" Surgeon-General Woodworth, ........ 353
" Heman P. Babcock, M. D ........ 235
" Dr. James Ives, ........ 472
Doremus, C. A. Prof., M. D. Chemical Analysis in cases of Obscure Diagnosis, ........ 281
Davis, H. S Clergyman's Sore Throat, ........ 302
Drugs, Adulteration of ........ 389
Disinfectant, New, Cheap and Self-generating, ........ 66
Dressing, Antiseptic. By Prof. Lister ........ 61
Diehl, C., M. D, Renal Calculus, ........ 416
Druggists, State Association of ........ 435
Dispensaries, Free ........ 388
Dog, Biliary Secretion of, with reference to the Action of Cholagogues, ........ 386
Doctors, Mortality of ........ 183
Doctors, Eulogium on ........ 232

### E

Eye and Ear Infirmary, or Oculist and Aurist ........ 353
Edison's Ear-trumpets, ........ 113
Eames, C. W., Dermoid and Ovarian Cysts, 98
Exchange Journals. List of ........ 195
Epidemic. Yellow Fever ........ 114
English Quarantine, ........ 232
Empyema and Pleurisy, Treatment of ........ 26
Editor Journal of Insanity, Libel suit against the ........ 151
Elsburg. Dr., Laryngology by ........ 394

### F

Fowler, Joseph, M. D., Rachitis, ........ 81
" " M. D., Cancerous Disease of the Lungs ........ 41
Ford, Willis E., M. D. Pulmonary Consumption, ........ 161

Free Dispensaries, 388
Femur, Amputation of 369
Fistula in Ano. By T. G. Comstock, M. D., 294

**G**

Gynæcological Society American, Annual Meeting of 142
Grissom's, Dr., Charges against Dr. Hammond, 188
Gross, Prof. S. D., Complimentary Dinner to 377
Glycerite of Animal Hypophosphites. By Elwood C. Lester, M. D., 13

**H**

Hamilton, Prof. Frank H., Warm and Hot Water as Surgical Dressing, 328
Hernia, Umbilical, in the Adult. By W. W. Potter, M. D., 200
Health Board, Proposed National, 230
"Happy New-Year," 233
Hamilton on Nervous Diseases, 194
Hygiene, Rural, 36
Hutchinson, Prof, Joseph C., M. D. Mechanical Treatment of Hip, Knee and Ankle-joints, 418

**I**

Items 35, 74, 154, 193
Important Medical Works for Publication, 155
Iron, Dialyzed, 181, 394
"Index Medicus," 234
Iodoform, Therapeutic value of 266
Insanity, Early Indications of. By Judson B. Andrews, M. D., 241
Iodide of Potassium in Syphilis. By J. F. Miner 173
Insane Asylums, Employment of Female Assistant Physicians in Female Wards of 391
Insane Asylum Investigation, Petition for 470
Insame Asylum, Appointments to Utica, 152
Illinois State Board of Health, attacking Quackery, 184
Ives, Dr. James, Death of 471

**J**

Joint, Anatomy and Dislocation of. By Wm. F. Sheehan, M. D,, 361
Journals, New 277
Journals, List of Exchange 195
Joints, Mechanical Treatment of the Hip, Knee and Ankle-joints, with cases and illustrations. By Jos. C. Hutchinson, M.D. 418

**L**

Leonard's, C. Henri, Books, 152
Libel Sut against the Editor of the American Journal of Insanity, 151
Laryngology. By Dr. Elsburg, 394
Lactopeptine, 72, 278, 436
Letter to the Journal, 190
Lister, Prof., Antiseptic Dressing of 61

**M**

Mills, Henry, Causes of Diphtheria by 366
Medicinal Wines, 354
Maltine, and its Combination, 278
Maltine, 193, 354
Medicine and Hygiene., 274
Medical Colleges, Call for Convention of 319
Medical Colleges, Convention of 433
Medical Editors, American Association of 433
Medical Society of the County of Albany 219 259
Medical Act, Wisconsin, 469
Malpractice in Maine, Suits for. 21
Malpractice, Suits for, so common as to constitute an epidemic, 192
Mortality of Doctors, 183
Metric System in a Nut-Shell, 19
Metric System, 73
Miner, Prof. J. F. Iodide of Potassium in Syphilis. 173
" " Extirpation of the Spleen, 367
" " Cases in private practice of 10
" " Removal of Ovarian Tumor, 10
" "Ascites, Paracentisis Abdominatis, 11
" " Polypus of Uterus, 12
" " Atresia Vagina, 12
Mercury, Physiological and Therapeutic properties of 28
Morphia, Observations on Deep Injection of 59
Milk in Bright's Disease, Intravenous injection of, 464

**N**

Newport, R. I., Scarlet Fever in 389
Navy, Surgeon-General of the 185
Nitrate of Silver as a Uterine Caustic 187
Nervous Diseases, Hamilton on 194
New Nerve 229

**O**

Ozone, Poisonous nature of 272
Open Air Treatment, 73
Office Card 471

**P**

Plague, Yellow Fever 67
Pleurisy and Empyemia, Treatment of 25
Peterson, Fred. Renal Calculus. Reported by 416
Pancreas, Scirrhus of 121
Pruritis Vulvæ and Diabetes 31
Popular Science Monthly 34
Physicians, are they to be driven back to furnishing their own medicines? 73
Physicians, list of, who have died of Yellow Fever in Memphis 186
Potter, W. W., M. D., Umbilical Hernia in the adult. by 200
Perry, T. K., M. D. Report of Medical Society of the County of Albany, by 219, 259
Prolapsus Ani, 370
Paracentesis Thorasis 130

**Q**

Quarantine 109
Quarantine, English 232
Quackery, Illinois State Board of Health, attacking 184

**R**

Rachitis. By Joseph Fowler, M. D., ........ 81
Religion and Chloroform........................ 354
Rochester, T. F., M. D. Treatment of Ulcers with saturated solution Chlorate Potassium, by........................................ 177
Rochester, Prof. T. F., Address by........ 441
Rogers, Henry W. Extirpation of the Spleen, reported by.................... 367
Renal Calculus, service of Dr. C. Diehl..... 416
Resolutions, Board of Health,............... 390

**S**

Sheehan, Wm. F., M. D. The anatomy and dislocation of the shoulder-joint, by 361
Scarlet Fever in Newport, R. I.,............ 389
Surgeon-General of the Navy ............... 185
Science in Vanderbilt University,........... 32
Smartweed........................................ 66
Shellac Cloth or Poroplastic Spinal Jacket, application of .............................. 274
Surgeon-General Woodworth,................ 353
Sewer-gas and Diphtheria..................... 113
Suits against Surgeons. By Geo. M. Blake, M. D.,..................................... 309
Shank, Dr. A. R. Vital Force, by........... 1
Storer, Dr. H. R. Protest from............. 117
Stoddard, Prof. E. V., letter from........... 456

**T**

Tumors, Malignancy in, by P. W. Van Peyma, M. D.......................................... 401
Telephonic Exchange, Buffalo................ 354
Telephonic Communication.................. 277
Telegraphists Cramp.......................... 29

**U**

University of Buffalo, Commencement in Medical Department of.................... 316
Uterine Polypi and Fibroids................ 371
Uterine Caustic, Nitrate of silver as a...... 187
Ulcers, treatment of, with saturated solution Iodide Potassium. By T. M. Rochester, ............................................ 177
Uranine............................................ 235
Umbilical Hernia, by W. W. Potter, M. D. 200
United States Census, letter from Joseph K. Barnes, Surgeon-General U. S. Army.... 226

**V**

Van Peyma, P. W., M. D., Malignancy in Tumors, by ........................................ 401
Vanderbilt University. Science in......... 32
Vital Force, by A. R. Shank, M. D.,......... 1
Vienna, letter from............................ 460
Vol. XVIII Buffalo Medical and Surgical Journal ........................................ 33
Vol. XVIII, Completion of, and future of the Journal,...................................... 471

**W**

Waddel, Dr. Death of.......................... 394
Woodworth, Surgeon-General ............... 353
Warm and Hot water as Surgical Dressing, By Prof. Frank H. Hamilton............... 328
White, Prof. James P. Prospects and Progress of the Buffalo Medical College, by.. 321
White, Prof. James P. Post-partum Hemorrhage, by,................................... 411
Wisconsin Medical Act ........................ 469
Wall, C. A., B. S., M. D. Surgical Cases in the practice of J. F. Miner, reported by.. 10
56, 173.
Waid, J. F., M. D. Scirrhus of the Pancreas, by........................................ 121
Wanga plant and Voudooism................ 67

**Y**

Yellow Fever Epidemic....................... 114

Yellow Fever Plague.......................... 67

S.G.O.L.
INDEXED

### THE BUFFALO
### MEDICAL AND SURGICAL
### JOURNAL.

Wisconsin Medical Act,.................. .. 469

EDITORIAL.

MEDICAL AND SURGICAL JOURNAL.

PUBLISHED MONTHLY.—Containing Original Articles, Reports of Medical Societies and Hospitals, Editorial, Reviews, Correspondence, Army News, etc., etc; including the usual variety of Medical Periodical Publications. Specimen copies sent on application to BUFFALO MEDICAL AND SURGICAL JOURNAL, J. F. MINER, M. D., 978 Main Street, BUFFALO, N. Y.

TERMS OF SUBSCRIPTION, $3 00 PER YEAR, IN ADVANCE,—All communications for publication should be sent before the fifteenth (15) of each month, or they will of necessity lie over until the next number. No change can be made in advertisements after the twenty-fifth.

Correspondence and original articles are solicited. Publication is no evidence that the views of the author are endorsed by the Journal.

